# Dose-response association of obesity and risk of mental health among tehranian residents: result of a cross-sectional study

**DOI:** 10.1186/s12889-024-18670-z

**Published:** 2024-05-29

**Authors:** Aliyu Tijani Jibril, Bahareh Jabbarzadeh Ganjeh, Amin Mirrafiei, Mahsa Firouzi, Reyhane Norouziasl, Shadi Ghaemi, Negar Bafkar, Ahmad Jayedi, Kurosh Djafarian, Sakineh Shab-Bidar

**Affiliations:** 1 Department of Community Nutrition, School of Nutritional Sciences and Dietetics, Tehran University of Medical Sciences, Tehran, Iran; 2 Social Determinants of Health Research Center, Semnan University of Medical Sciences, Semnan, Iran; 3 Department of Clinical Nutrition, School of Nutritional Sciences and Dietetics, Tehran University of Medical Sciences, Tehran, Iran; 4 Sports Medicine Research Center, Neuroscience Institute, Tehran University of Medical Sciences, Tehran, Iran; 5 Sports Medicine Research Center, Neuroscience Institute, Department of Community Nutrition, School of Nutritional Sciences and Dietetics, Tehran University of Medical Sciences, Tehran, Iran

**Keywords:** Mental disorders, Anxiety, Depression, Stress, Obesity, Body mass index, Body adiposity index, A body shape index

## Abstract

**Background:**

Obesity and mental health issues are two of the most prevalent global public health issues for a significant portion of people. The purpose of this study was to investigate the relationship between obesity indicators and mental health in Tehran-dwelling Iranian adults.

**Methods:**

We conducted a cross-sectional study on healthy Iranian adults using a convenience sampling technique. The short form of the Depression Anxiety and Stress Scale (DASS-21) was used to measure the outcome, and independent variables included body mass index (BMI), waist-to-hip ratio (WHR), waist-to-height ratio (WHtR), body adiposity index (BAI), and a body shape index (ABSI). The relationship between obesity and mental health was investigated using a multivariate logistic regression model. The non-linear dose-response relationships were evaluated using restricted cubic splines (RCS) with three knots. The Benjamini-Hochberg procedure was used to adjust for multiple testing.

**Results:**

In our study of 434 participants, females made up 52% of the participants, with a mean age of 38.57 years. In all, 54.6%, 53.9%, and 56.6% were classified as having anxiety, depression, and stress respectively. Logistic regression analysis showed that the odds of mental health components including anxiety, depression, or stress was not significantly different across the tertiles of the obesity indicators. We observed a significant dose-response relationship between BAI and ABSI and the risk of anxiety (P_Benjamini-Hochberg_ 0.028 > P_dose-response_ 0.023) and stress (P_Benjamini-Hochberg_ 0.028 > P_dose-response_ 0.003) but not depression (P_Benjamini-Hochberg_ 0.014 < P_dose-response_ 0.018). The lowest risk for anxiety was observed in people with a BAI of 28% and ABSI equal to 0.079. The risk of stress seemed to increase beyond an ABSI of 0.086.

**Conclusion:**

Our findings showed no direct linear association between obesity indices and anxiety. However, a dose-response relationship was observed between BAI and ABSI and the risk of anxiety and stress, indicating the need for further investigation.

**Supplementary Information:**

The online version contains supplementary material available at 10.1186/s12889-024-18670-z.

## Background

One billion people worldwide are estimated to suffer from a mental illness, according to a recent survey [1]. The prevalence of mental illness in the WHO Eastern Mediterranean Region, which includes several Middle Eastern countries, was found to be 10.3% for current prevalence, 4.5% for lifetime prevalence, and 6.0% for period prevalence. In the WHO Eastern Mediterranean Region, which includes various Middle Eastern countries, the current prevalence of mental illness was found to be 10.3%, lifetime prevalence was 4.5%, and period prevalence was 6.0% [2]. These rates are expected to rise due to ongoing wars and conflicts [3]. Depression and anxiety disorders are the most common mental illnesses in the Middle East [2, 3]. Mental disorders in the region were associated with 11.9 million disability-adjusted life years (DALYs) from 1990 to 2013, with Palestine, Djibouti, and Somalia recording the highest [3]. The prevalence of mental health disorders has increased in Iran, as it has in many other countries. According to estimates, there were roughly 20.0% mental health disorders among adults aged 15 and older in 1999 [4] and 23.4% in 2015 [5]. Furthermore, a recent population-based study carried out in Iran between December 2019 and February 2020 on 24,584 adults over the age of 15 discovered that the prevalence of mental disorders was 29.7% [6], indicating a trend in the upward direction over time. Accumulating evidence shows the link between mental and physical health. The fact that physical health may affect mental health and vice versa is becoming more widely recognized [7]. Numerous factors, including an individual’s body composition (being overweight or obese) [8–11], living with chronic diseases [12], and pandemic diseases such as COVID-19 [13], have been implicated as risk factors for developing mental disorders.

Obesity is a major public health concern, and it is affecting both developed and developing countries [14–16]. On a global scale, overweight and obesity were responsible for approximately 120 million disability-adjusted life years (DALYs) and 4.0 million deaths in 2015 [17]. Obesity prevalence tripled between 1975 and 2016, with 13% of adults globally (11% of males and 15% of women) obese in 2016 [18]. Obesity prevalence in Middle Eastern countries was estimated to be 21.17% in a recent comprehensive review and meta-analysis [19]. According to the combined estimate, 22.41% of Iranians were obese. The prevalence of obesity decreased from 22.41% (from 2000 to 2006) to 17.74% (from 2007 to 2013) according to trend analysis. However, the prevalence rose to 25.98% between 2014 and 2020 [19].

For a considerable number of people, obesity and mental health disorders co-occur, making them two of the most common global public health challenges [20, 21]. Obesity is a condition in which there is an abnormal or excessive accumulation of fat in the body, which can be harmful to one’s health. However, relying solely on the body mass index (BMI) to determine obesity may not accurately reflect the diverse characteristics of the population affected by this condition. BMI does not differentiate between fat mass and lean body mass, which can lead to overestimating fat in muscular athletes while underestimating muscle loss in older individuals [22, 23].

New anthropometric indices are being constantly developed to accurately predict body adiposity. The most notable of these are the body adiposity index (BAI) and the body shape index (ABSI).

ABSI, a novel index calculated using waist circumference, BMI, and height to predict visceral fat, was developed by Krakauer and Krakauer [24] with reported superiority in comparison to BMI and waist circumference (WC) for predicting morbidity and mortality. While a study showed that ABSI was not a good index for identifying morbidity-related risk factors [25], some researchers have shown that ABSI is closely related to metabolic diseases [26, 27]. Using WC and height, Bergman and colleagues proposed another novel tool, the body adiposity index (BAI), for evaluating adiposity [28]. With few reports that have confirmed the usefulness of BAI in the Asian population [29–31], several studies elsewhere have reported conflicting results [32–39].

Studies on the relationship between obesity and mental health status have been the subject of conflicting reports [8–11, 40–43]. Additionally, few studies have assessed this relationship using new anthropometric measures as indicators of obesity [44, 45], and to the best of our knowledge, there are no published reports on the association of obesity with mental health in any population comparing multiple novel anthropometric indices in a dose-response manner. Therefore, this study aimed to investigate the dose-response association between obesity and mental disorders (anxiety, depression, and stress) in Iranian adults.

## Methods

### Study design and selection of participants

This cross-sectional study was conducted among apparently healthy Iranian subjects between the ages of 20 and 59 years using a convenience sampling technique. These subjects were all residents of Tehran. Furthermore, pregnant and lactating women, diabetics, cancer patients, patients with cardiovascular disease, rheumatoid arthritis, chronic liver disease, and liver disease patients, as well as subjects with a history of stroke and heart attack, were not included in the study. Additionally, subjects suffering from Alzheimer’s and Parkinson’s disease, as well as subjects on special diets, were not included.

### Sample size determination

To calculate sample size, we used a 40% prevalence of mental health disorders with a power of 95% and an error margin of 5% using an online sample size calculator: http://www.raosoft.com/samplesize.html. The 40% prevalence estimate used in the sample size calculations in this study was based on the results of a previous investigation of the prevalence of mental health disorders among Iranian adults [46]. Based on this method, we needed 363 subjects for this study.

### Mental health

To measure the outcome, we used the Depression, Anxiety, and Stress Scale (DASS-21) in its short form. This self-report scale is specifically designed to evaluate the core symptoms of common mental health disorders, such as depression, anxiety, and stress. It comprises 21 items, with seven items allocated to each domain. Participants assess their experience over the past week and rate each item on a four-point scale. Using the Likert scoring system of 0-1-2-3, scores range from 0 to 63, with lower scores indicating better mental health status [47].

The factor structure and psychometric properties of the Persian version of the Depression Anxiety Stress Scale-21 (P-DASS-21) have been investigated and confirmed as a valid and reliable tool for evaluating depression, anxiety, and stress in non-clinical Iranian samples [47, 48]. The study [47] established the validity of the P-DASS-21 in three ways: confirmatory factor analysis, convergent validity, and discriminant validity. The confirmatory factor analysis tested the adequacy of the three-factor solution of the DASS-21 and yielded an acceptable descriptive fit based on predetermined cutoffs. The DASS-21 scales’ convergent validity was investigated by comparing them to other measures known to measure similar constructs and substantial positive correlations were discovered. The discriminant validity of the DASS-21 Anxiety and Stress scales was examined by comparing diagnostic categories, and the results supported the hypotheses that patients with mood disorders, Generalized Anxiety Disorder (GAD), and Obsessive-Compulsive Disorder (OCD) would score significantly higher on the Anxiety and Stress scales than non-clinical individuals. Furthermore, the P-DASS-21 has been demonstrated to have adequate internal consistency, with Cronbach alpha values of 0.85, 0.85, and 0.94 for depression, anxiety, and stress, and 0.94 for the total DASS-21 score [47]. The intra-class correlation coefficients ranged from 0.77 to 0.89 [47] indicating strong test-retest reliability. Therefore, the DASS-21 has been validated and found to be a reliable tool for evaluating depression, anxiety, and stress in Iranian populations.

## Anthropometrics

Body weight was measured with light clothes without shoes using a digital scale (Seca 808, Germany) to the nearest 0.1 kg. Height was measured using a stadiometer (Seca, Germany) to the nearest 0.1 cm. Waist circumference, hip circumference, and neck circumference (all in centimeters) were assessed using non-stretchable tape measures to the nearest 0.1 cm. We then computed BMI by dividing weight (kg) by height (m) squared [49]. WHR was calculated by dividing waist circumference (cm) by hip circumference (cm). WHtR, on the other hand, was calculated by dividing waist circumference (cm) by the individual’s height (cm). BAI and ABSI were calculated by using height, waist and hip circumference, and BMI as below [28, 29, 50]:

$${\rm{AI}}{\mkern 1mu} {\rm{ = }}{\mkern 1mu} {{{\rm{Hip}}} \over {{\rm{height}}\sqrt {{\rm{height}}} }}{\rm{ - 18}}$$

$${\rm{body}}\,{\rm{shape}}\,{\rm{index}}\,({\rm{ABSI}})\, = \,{{{\rm{WC}}} \over {{\rm{BM}}{{\rm{I}}^{{\rm{2/3}}}}{\rm{*heigh}}{{\rm{t}}^{{\rm{1/2}}}}}}$$

### Covariates

To evaluate the factors that could affect the outcome, we collected socio-demographic data such as age, gender, education, marital status, smoking habits (individuals who have never smoked or have stopped smoking were classified as never/ former smokers, whereas those who currently smoke were classified as smokers), medical history, use of drugs or supplements, and menopause status. Additionally, we utilized the International Physical Activity Questionnaire (IPAQ) to measure the physical activity level of the subjects. We categorized the participants into three groups based on their activity level: those who did not engage in any physical activity, those who were moderately active (1–3 h per week), and those who were active (more than 3 h per week). The International Physical Activity Questionnaire (IPAQ) has been translated and validated in the Iranian population, with studies reporting good reliability and validity of the questionnaire [51] among healthy Iranian adults. The result of the study indicated that all domains of the questionnaire met the minimum reliability standards (intra-class correlation [ICC] > 0.7), except for Leisure-time physical activity [51].

### Statistical analysis

The STATA Statistical Software (StataMP 15. Stata Corp., College Station, TX: StataCorp LP) was used to perform all statistical analyses. The Q-Q plot and the Kolmogorov-Smirnov test were used to determine the data’s normality. The comparison between mental health’s status was done by independent t-test and χ2 tests for continuous and categorical variables, respectively. To investigate the independent relationship between obesity and mental health and to account for potential confounding variables, a multivariate logistic regression model was used. The univariate model was unadjusted. We adjusted for potential covariates including age, sex, educational level, and marital status in model 1. Model 2 was further adjusted for smoking status, occupation, living status, diabetes, and physical activity level. To analyze the trend of ORs for BMI, WHR, WHtR, BAI, and ABSI, each of the variables was analyzed as tertiles in the logistic regression analysis.

We applied restricted cubic splines (RCS) with three knots to determine non-linear relationships in regression models between mental health and BMI, WHR, WHtR, BAI, or ABSI. The RCS models were adjusted for age, sex, education level, marital status, smoking, occupation, living status, diabetes, and physical activity. To account for multiple tests, we applied the Benjamini-Hochberg procedure [52] and set a 2-sided 5% alpha error threshold.

### Ethics statement

Those who met the criteria and agreed to participate in the study were included and signed informed consent. The study protocol was approved by the Research Ethics Committee of Tehran University of Medical Sciences (Ethics No: *IR.TUMS.MEDICINE.REC.1401.325*).

## Results

The general characteristics of the study participants, divided into their mental health status, are listed in Table 1. Overall, we included a total of 434 subjects in our study, of whom 237 (54.6%), 234 (53.9%), and 246 (56.6%) were classified as having anxiety, depression, and stress respectively. 52% of our study participants were female. The mean age of our subjects was 38.57 years with a standard deviation of 9.67 years. We observed statistically significant differences between subjects with and without anxiety based on their gender (*P* = 0.02), level of education (*P* = 0.007), and employment status (*P* = 0.001). Subjects grouped under stress were significantly different in terms of age and gender (*P* = 0.006), marital status (*P* = 0.04), level of education (*P* = 0.02), employment (*P* = 0.004), and smoking status (*P* = 0.03). The mean of the anthropometric characteristics was not statistically significantly different between any of the groups.

**Table 1:** General characteristics of study participants across mental health status

	Anxiety		Depression		Stress	
	Yes (237)	*P*-value	Yes (234)	*P*-value	Yes (246)	*P*-value
Age (years)	38.77 ± 9.60^a^	0.63 ^b^	39.04 ± 9.63^a^	0.27 ^b^	39.67 ± 9.55^a^	0.006 ^b^
Gender (%) Male Female	60.1 49.6	0.02 ^c^	58.7 49.6	0.05 ^c^	63.5 50.4	0.006 ^c^
Marital status (%) Married with partner Never married Widowed/divorced/separated	55.5 53.4 47.8	0.74 ^c^	57.3 49.6 34.8	0.06 ^c^	60.9 49.6 43.5	0.04 ^c^
Education level (%) Less than 11th grade High school graduate College graduate or above	11.1 35.3 56.3	0.007 ^c^	22.2 47.1 54.8	0.12 ^c^	11.1 58.8 57.5	0.02 ^c^
Employment (%) Employed Unemployed	59.2 39.4	0.001 ^c^	56.4 45.5	0.05 ^c^	60.7 44.4	0.004 ^c^
Smoking (%) Yes No	55.0 48.3	0.48 ^c^	55.3 34.5	0.03 ^c^	58.4 37.9	0.03 ^c^
Physically active (%) Low Moderate High	51.1 59.9 56.2	0.27 ^c^	52.7 56.5 53.9	0.79 ^c^	53.3 59.9 57.3	0.47 ^c^
Weight (kg)	76.78 ± 14.04^a^	0.30 ^b^	76.30 ± 13.93^a^	0.76 ^b^	77.06 ± 14.04^a^	0.12 ^b^
Height (cm)	168.11 ± 9.10^a^	0.07 ^b^	167.89 ± 9.05^a^	0.22 ^b^	168.00 ± 9.00^a^	0.11 ^b^
Waist circumference (cm)	92.51 ± 12.12^a^	0.95 ^b^	92.55 ± 12.94^a^	0.99 ^b^	92.80 ± 12.19^a^	0.65 ^b^
Hip circumference (cm)	105.05 ± 7.51^a^	0.77 ^b^	104.79 ± 8.07^a^	0.34 ^b^	104.85 ± 7.71^a^	0.39 ^b^
WHR	0.88 ± 0.08^a^	0.51 ^b^	0.88 ± 0.09^a^	0.41 ^b^	0.88 ± 0.08^a^	0.16 ^b^
WHtR	0.55 ± 0.07^a^	0.87 ^b^	0.55 ± 0.08^a^	0.76 ^b^	0.55 ± 0.07^a^	0.98 ^b^
BMI (kg/m²)	27.16 ± 4.47^a^	0.82 ^b^	27.07 ± 4.55^a^	0.85 ^b^	27.27 ± 4.37^a^	0.41 ^b^
ABSI (m^11/6^kg^−2/3^)	0.08 ± 0.005^a^	0.38 ^b^	0.08 ± 0.005^a^	0.87 ^b^	0.08 ± 0.005^a^	0.30 ^b^
BAI (%)	30.47 ± 5.46^a^	0.16 ^b^	30.48 ± 5.77^a^	0.19 ^b^	30.46 ± 5.46^a^	0.13 ^b^

^a^means ± standard deviation (SD)

^b^Obtained from T-test for continuous variables

^c^Obtained from chi-square test for categorical variables

BMI, Body Mass Index; ABSI, A Body Shape Index; BAI, Body Adiposity Index

Table 2 shows the results of a logistic regression analysis investigating the relationship between mental health status (anxiety, depression, and stress) and BMI, WHR, WHtR, BAI, and ABSI classified by tertiles. There were no significant differences in the odds of anxiety, depression, or stress across the tertiles of obesity indicators. These non-significant effects remained even after adjusting for potential covariates including age, sex, education level, marital status, smoking, occupation, living status, diabetes, and physical activity. The adjusted p-values (using the Benjamini-Hochberg procedure) in the final model confirmed the non-significant results.

**Table 2:** Logistic regression analyses of the relationship between BMI, WHR, WHtR, BAI, ABSI and anxiety (classified by tertiles)

Variable	Univariate		Model 1		Model 2		
	OR (95% CI)	*P*-value	OR (95% CI)	*P*-value	OR (95% CI)	*P*-value	*P*_B−H_
**Anxiety**							
BMI tertiles Lowest Middle Highest P for trend	1.15 (0.72–1.85) 1.14 (0.71–1.83) 1.00 0.81	0.56 0.59	1.05 (0.63–1.75) 1.22 (0.74-2.00) 1.00 0.73	0.85 0.43	1.02 (0.58–1.80) 1.31 (0.77–2.23) 1.00 0.56	0.94 0.32	0.12 0.07
WHR tertiles Lowest Middle Highest P for trend	1.10 (0.68–1.78) 1.11 (0.69–1.79) 1.00 0.88	0.69 0.65	0.55 (0.29–1.06) 0.81 (0.47–1.37) 1.00 0.18	0.07^†^ 0.42	0.51 (0.25–1.05) 0.65 (0.35–1.18) 1.00 0.18	0.06^†^ 0.15	0.01 0.05
WHtR tertiles Lowest Middle Highest P for trend	0.89 (0.55–1.44) 0.84 (0.52–1.35) 1.00 0.76	0.64 0.46	0.78 (0.46–1.33) 0.92 (0.56–1.51) 1.00 0.64	0.35 0.73	0.87 (0.49–1.56) 0.98 (0.56–1.70) 1.00 0.89	0.64 0.93	0.10 0.11
ABSI tertiles Lowest Middle Highest P for trend	0.85 (0.53–1.37) 0.83 (0.51–1.35) 1.00 0.71	0.49 0.45	0.62 (0.36–1.08) 0.74 (0.44-1 23) 1.00 0.23	0.09 0.23	0.63 (0.35–1.14) 0.61 (0.35–1.07) 1.00 0.20	0.12 0.08	0.04 0.02
BAI tertiles Lowest Middle Highest P for trend	0.76 (0.47–1.22) 0.82 (0.51–1.33) 1.00 0.50	0.25 0.42	1.21 (0.64–2.27) 1.01 (0.59–1.73) 1.00 0.79	0.55 0.96	1.19 (0.59–2.38) 1.00 (0.56–1.79) 1.00 0.84	0.62 0.99	0.08 0.14
**Depression**							
BMI tertiles Lowest Middle Highest P for trend	1.03 (0.64–1.65) 1.01 (0.63–1.62) 1.00 0.99	0.91 0.95	0.84 (0.50–1.40) 1.05 (0.64–1.71) 1.00 0.68	0.50 0.85	0.80 (0.46–1.39) 1.10 (0.65–1.86) 1.00 0.52	0.42 0.72	0.08 0.12
WHR tertiles Lowest Middle Highest P for trend	1.21 (0.75–1.95) 1.324(0.82–2.13) 1.00 0.50	0.44 0.24	0.74 (0.39–1.40) 1.09 (0.65–1.84) 1.00 0.36	0.35 0.75	0.73 (0.36–1.47) 0.95 (0.53–1.69) 1.00 0.61	0.38 0.86	0.07 0.14
WHtR tertiles Lowest Middle Highest P for trend	0.79 (0.49–1.28) 1.03 (0.64–1.65) 1.00 0.51	0.34 0.90	0.59 (0.32-1.00) 1.08 (0.66–1.78) 1.00 0.05	0.05^†^ 0.75	0.67 (0.38–1.20) 1.19 (0.69–2.05) 1.00 0.13	0.17 0.53	0.01 0.04
ABSI tertiles Lowest Middle Highest P for trend	0.98 (0.61–1.59) 1.47 (0.91–2.38) 1.00 0.17	0.94 0.11	0.80 (0.47–1.37) 1.37 (0.83–2.26) 1.00 0.11	0.41 0.22	0.85 (0.47–1.52) 1.34 (0.78–2.33) 1.00 0.24	0.58 0.29	0.11 0.04
BAI tertiles Lowest Middle Highest P for trend	0.67 (0.42–1.09) 0.87 (0.54–1.41) 1.00 0.25	0.10 0.58	0.67 (0.36–1.25) 0.82 (0.49–1.40) 1.00 0.46	0.20 0.47	0.62 (0.31–1.24) 0.83 (0.47–1.48) 1.00 0.40	0.17 0.53	0.01 0.10
**Stress**							
BMI tertiles Lowest Middle Highest P for trend	1.60 (0.99–2.58) 1.25 (0.77–2.02) 1.00 0.16	0.05^†^ 0.36	1.32 (0.79–2.22) 1.35 (0.82–2.23) 1.00 0.44	0.28 0.24	1.30 (0.74–2.30) 1.41 (0.82–2.44) 1.00 0.45	0.36 0.21	0.10 0.05
WHR tertiles Lowest Middle Highest P for trend	1.49 (0.92–2.42) 1.03 (0.63–1.66) 1.00 0.19	0.10 0.91	0.78 (0.41–1.49) 0.73 (0.43–1.26) 1.00 0.52	0.44 0.25	0.71 (0.35–1.46) 0.62 (0.34–1.14) 1.00 0.31	0.35 0.12	0.08 0.02
WHtR tertiles Lowest Middle Highest P for trend	1.10 (0.68–1.78) 1.00 (0.62–1.62) 1.00 0.90	0.70 1.00	0.85 (0.50–1.45) 1.06 (0.64–1.76) 1.00 0.70	0.55 0.82	0.97 (0.54–1.74) 1.19 (0.68–2.90) 1.00 0.75	0.92 0.54	0.14 0.11
ABSI tertiles Lowest Middle Highest P for trend	0.90 (0.55–1.45) 0.94 (0.58–1.52) 1.00 0.90	0.66 0.79	0.63 (0.37–1.10) 0.79 (0.47–1.31) 1.00 0.26	0.10 0.35	0.62 (0.34–1.12) 0.69 (0.39–1.21) 1.00 0.25	0.11 0.19	0.01 0.04
BAI tertiles Lowest Middle Highest P for trend	0.91 (0.56–1.47) 0.87 (0.54–1.41) 1.00 0.84	0.68 0.57	1.43 (0.75–2.72) 1.02 (0.60–1.76) 1.00 0.44	0.27 0.93	1.46 (0.72–2.97) 1.05 (0.59–1.89) 1.00 0.53	0.29 0.86	0.07 0.12

*Notes* Model 1: Adjust for age, sex, education level and marital status. Model 2: Adjust for age, sex, education level and marital status, smoking, occupation, living status, diabetes, and physical activity

BMI, Body Mass Index; WHR, Waist-to-Hip Ratio; WHtR, Waist-to-Height Ration; ABSI, A Body Shape Index; BAI, Body Adiposity Index

^†^Marginally significant

*P*_B−H_: *P*-value adjusted using the Benjamini-Hochberg procedure

*p* for trend; *p*-value from Test for Trend

**Fig. 1:**
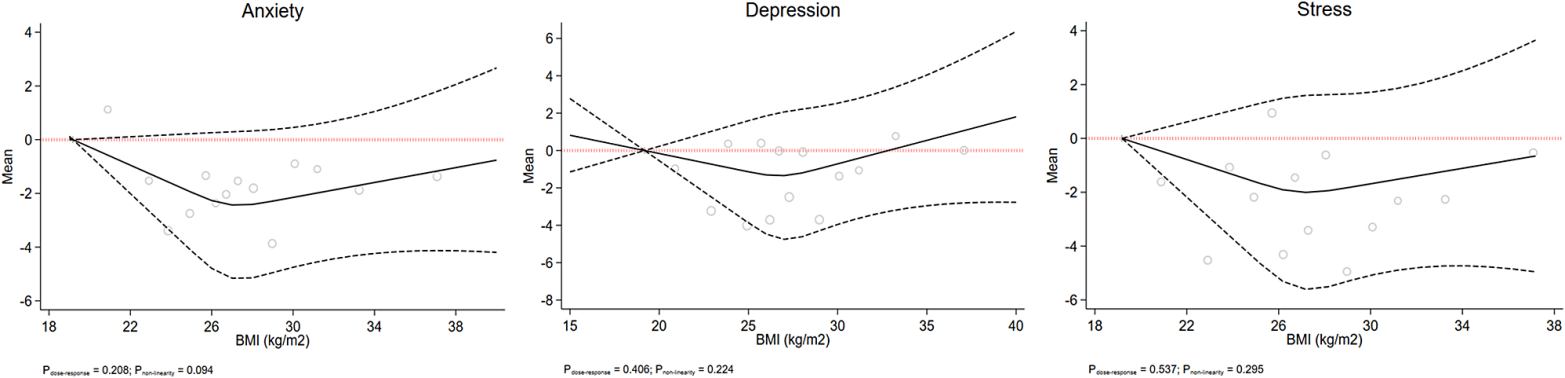
The dose-response association of BMI with mental health status. The red dash lines indicates the knots, the black dash lines represents the lower and upper confidence intervals, the solid black line shows the estimate

**Fig. 2:**
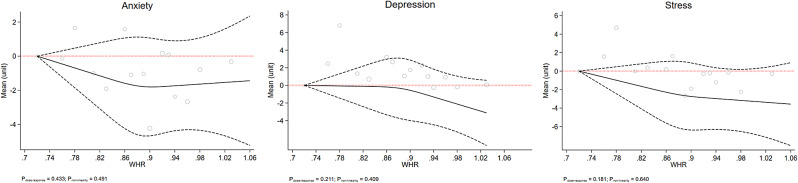
The dose-response association of WHR with mental health. The red dash lines indicates the knots, the black dash lines represents the lower and upper confidence intervals, the solid black line shows the estimate

**Fig. 3:**
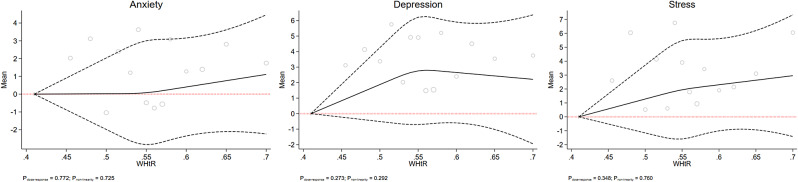
The dose-response association of WHtR with mental health. The red dash lines indicates the knots, the black dash lines represents the lower and upper confidence intervals, the solid black line shows the estimate

**Fig. 4:**
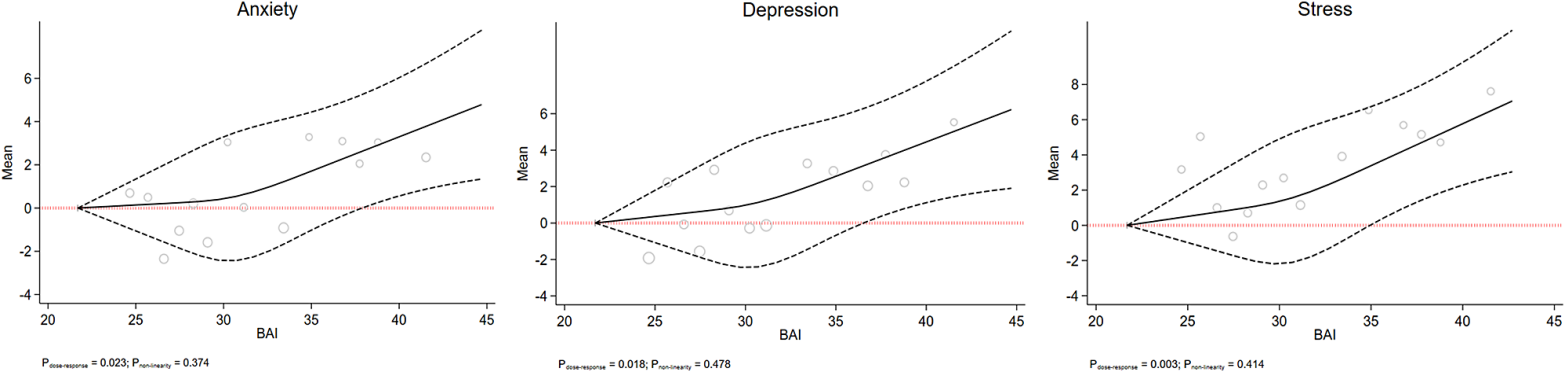
The dose-response association of BAI with mental health. The red dash lines indicates the knots, the black dash lines represents the lower and upper confidence intervals, the solid black line shows the estimate

**Fig. 5:**
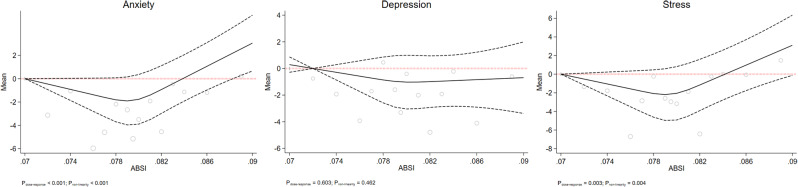
The dose-response association of ABSI with mental health. The red dash lines indicates the knots, the black dash lines represents the lower and upper confidence intervals, the solid black line shows the estimate

Figs. 1, 2, 3, 4 and 5 show the RCS models after adjusting for age, sex, education level, marital status, smoking, occupation, living status, diabetes, and physical activity. This study failed to observe any significant dose-response association between mental health status and BMI, WHR, or WHtR, which are depicted in Figs. 1 and 2, and 3, respectively. Fig. 4 shows a linear dose-response association between BAI and mental health. We observed a positive dose-response relationship between BAI and the risk of depression (P_dose−response_ = 0.018), anxiety (P_dose−response_ = 0.023), and stress (P_dose−response_ = 0.003). The results for the observed associations were further confirmed by the Benjamini-Hochberg procedure to handle multiple testing adjustments for anxiety (P_Benjamini−Hochberg_ 0.028 > P_dose−response_ 0.023) and stress (P_Benjamini−Hochberg_ 0.028 > P_dose−response_ 0.003) but not depression (P_Benjamini−Hochberg_ 0.014 < P_dose−response_ 0.018).

Fig. 5 also shows a non-linear does-response association between ABSI and mental health. Our study revealed a U-shaped, non-linear inverse dose-response association between ABSI and the risk of anxiety (P_dose−response_ < 0.001) and stress (P_dose−response_ = 0.003). We found that an ABSI value of around 0.079 indicated the lowest risk for stress, but the risk of stress seemed to increase beyond an ABSI of 0.086. We confirmed these findings using the Benjamini-Hochberg procedure for both anxiety (P_dose−response_; < 0.001 < P_Benjamini−Hochberg_ 0.014) and stress (P_dose−response_; 0.003 < P_Benjamini−Hochberg_ 0.028).

## Discussion

We observed no evidence of a direct linear association between obesity indices (BMI, WHR, WHtR, BAI, and ABSI) and the risk of mental health disorders among our study subjects. However, we found a dose-response association between anxiety and stress with ABSI and BAI.

There is disagreement over the link between obesity and mental health disorders as some studies show significant effects [53–57] while others do not [56, 58, 59].

Our study’s findings are consistent with earlier studies by Zedler et al. [56] and Grundy et al. [59]. These studies did not find a correlation between mental health disorders and obesity when using various measures such as WHtR and BMI. Additionally, Bruffaerts et al. found no significant association between mental disorders, including anxiety, and BMI [58]. In contrast to our results, a recent study from the Netherlands Mental Health Survey and Incidence Study-2 found obesity, based on BMI, to be a significant risk factor for the onset of anxiety disorders during a three-year follow-up period [53]. Other studies have also observed a significant association between WHR and WHtR and depression and anxiety, particularly among women [56, 57].

Meanwhile, other studies have also suggested a dose-response association between obesity and mental disorders. Ma et al. [60]. , found a significant U-shape correlation between BMI, WHtR, and depression, specifically, people with slightly overweight had the lowest risk of depression. Another meta-analysis of longitudinal studies found that obesity at baseline increased the risk of depression at follow-up, with a pooled odds ratio (OR) of 1.55 [61]. This association was stronger than the association between depression and overweight, suggesting a dose-response gradient [61]. Furthermore, a meta-analysis of 51 studies found that obesity was associated with an increased risk of anxiety, with the effect being stronger among women and in clinical samples [62].

Although the anthropometric indices did not show any linear association with the mental health status, similar to the above studies, we found a non-linear dose-response association between anxiety and stress and ABSI and BAI. According to previous reports, ABSI and BAI, as indicators of obesity and are more accurate predictors of different types of obesity than traditional indices [24, 28]. They haven’t, however, been widely applied in studies on mental health.

As far as we know, no previous research has explored the association between BAI and mental health disorders. Our study revealed a significant correlation between higher BAI scores and increased levels of anxiety, depression, and stress. The Benjamini-Hochberg procedure provided additional evidence supporting the dose-response relationship between BAI and anxiety and stress, but not depression.

To date, there have been limited investigations into the correlation between ABSI and mental well-being, and the results have been inconsistent [44, 45]. In research conducted by Lofti et al. [44], they observed that anxiety was linked to ABSI in women but not men. Previously, Hadi and colleagues [45] conducted a study that did not find any significant association between ABSI and anxiety. The reason for the disparity in findings among these studies could be due to differences in the study population, including sample size and participants. Hadi et al.‘s study [45] which had only 307 participants, focused solely on overweight and obese individuals, with a vast majority (approximately 81%) being female, possibly explaining the lack of correlation. In contrast, our study, similar to Lotfi et al.‘s [44] included participants with varying weight statuses and a larger sample size. Furthermore, we gathered and analyzed data on several potential confounding variables, such as education level, smoking habits, employment status, living situation, diabetes status, and physical activity level, in addition to age and gender, which Hadi et al. only considered.

Some potential biological and psychological factors might explain the observed relationship between the BAI and ABSI and anxiety and stress in our study. BAI and ABSI have a strong correlation with visceral fat [24]. Through a larger production of inflammatory cytokines, including C-reactive protein and interleukin 6, visceral adipose tissue may play a significant role in the link between BAI and ABSI and anxiety and stress [63, 64]. These inflammatory cytokines can increase oxidative damage and systemic inflammation [65]. The pathophysiology of anxiety has been linked to an increase in oxidative stress markers in the brain, such as glutathione and malondialdehyde, in overweight and obese individuals [66]. Chronic stress can also result in dysregulation of the hypothalamic-pituitary-adrenal axis, which results in higher cortisol levels and is linked to obesity and anxiety [67, 68]. Another potential contributing factor is dysregulation of the hypothalamic-pituitary-adrenal system, which would cause the corticotropin hormone to produce an excessive increase in cortisol and result in a persistent hypercortisolism state that causes abdominal obesity and anxiety symptoms [69]. Additionally, it has been suggested that stigmatization, weight discrimination, and a lack of social support may negatively impact obese people, causing psychological stress and the subsequent development of anxiety [70–72].

### Limitations

Our investigation has some limitations that need to be acknowledged. Firstly, as it is a cross-sectional study, we cannot establish a cause-and-effect relationship. Additionally, it is plausible that anxiety and stress symptoms may have emerged after the onset of obesity. Hence, it is advisable to exercise caution while extrapolating the results to a broader population. Lastly, there is a possibility that there are some unidentified factors that may have influenced the observed link.

Although our study has some limitations, it also has several advantages. It is the first to investigate the correlation between mental illness and obesity by comparing various traditional and novel anthropometric indices. We assessed mental health using validated questionnaires and had trained personnel evaluate the anthropometric traits. Additionally, we considered several potential confounding factors in our analysis and used the Benjamini-Hochberg method to adjust for multiple tests.

## Conclusion

Anxiety and stress showed a non-linear dose-response correlation with BAI and ABSI in the adult population of Iran, while no correlation was found with BMI, WHR, or WHtR. Therefore, we propose that further investigation should be conducted on BAI and ABSI in a larger sample size to confirm the causal relationship, determine their usefulness, and integrate them into the standard clinical assessment of anxiety.

### Electronic supplementary material

Below is the link to the electronic supplementary material.

Supplementary Material 1

## Data Availability

The datasets used during the current study are available from the corresponding author upon reasonable request.

## Abbreviations

DASS: Depression Anxiety and Stress Scale
WHR: Waist-to-hip ratio
WHtR: Waist-to-height ratio
BAI: Body adiposity index
ABSI: A body shape index
RCS: Restricted cubic splines
WC: Waist circumference
IPAQ: International Physical Activity Questionnaire

## References

[1] Health TLG (2020). Mental health matters. Lancet Global Health.

[2] Zuberi A, Waqas A, Naveed S, Hossain MM, Rahman A, Saeed K (2021). Prevalence of mental disorders in the WHO eastern mediterranean region: a systematic review and meta-analysis. Front Psychiatry.

[3] Ibrahim NK. Epidemiology of mental health problems in the Middle East. Handbook of healthcare in the Arab world. 2021:133 – 49.

[4] Noorbala AA, Yazdi SB, Yasamy M, Mohammad K (2004). Mental health survey of the adult population in Iran. Br J Psychiatry.

[5] Noorbala AA, Hajebi A, Faghihzadeh E, Nouri B (2017). Mental health survey of the Iranian adult population in 2015. Arch Iran Med.

[6] Noorbala AA, Maleki A, Yazdi SAB, Faghihzadeh E, Hoseinzadeh Z, Hajibabaei M (2022). Survey on mental health status in Iranian population aged 15 and above one year after the outbreak of COVID-19 disease: a population-based study. Arch Iran Med.

[7] Saxena S, Skeen S (2012). No health without Mental Health: challenges and opportunities in global mental health. Afr J Psychiatry.

[8] Khan QU, Zaffar S, Rehan AM, Rashid RR, Ashraf H, Hafeez F. Relationship of major depression with body mass index and salivary cortisol. Cureus. 2020;12(1). 10.7759/cureus.6577 PMC6999718 32047714

[9] van den Broek N, Treur JL, Larsen JK, Verhagen M, Verweij KJ, Vink JM (2018). Causal associations between body mass index and mental health: a mendelian randomisation study. J Epidemiol Community Health.

[10] Ul-Haq Z, Mackay DF, Fenwick E, Pell JP (2014). Association between body mass index and mental health among Scottish adult population: a cross-sectional study of 37272 participants. Psychol Med.

[11] Momtaz YA, Haron SA, Hamid TA, Ibrahim R, Tanjani PT (2018). Body mass index (BMI) and cognitive functions in later life. Curr Alzheimer Res.

[12] Smit F, Comijs H, Schoevers R, Cuijpers P, Deeg D, Beekman A (2007). Target groups for the prevention of late-life anxiety. Br J Psychiatry.

[13] Dragioti E, Li H, Tsitsas G, Lee KH, Choi J, Kim J (2022). A large-scale meta‐analytic atlas of mental health problems prevalence during the COVID‐19 early pandemic. J Med Virol.

[14] Collaboration NRF (2016). Trends in adult body-mass index in 200 countries from 1975 to 2014: a pooled analysis of 1698 population-based measurement studies with 19· 2 million participants. Lancet.

[15] Djalalinia S, Qorbani M, Peykari N, Kelishadi R (2015). Health impacts of obesity. Pakistan J Med Sci.

[16] Collaborators (2016). Global, regional, and national comparative risk assessment of 79 behavioural, environmental and occupational, and metabolic risks or clusters of risks, 1990–2015: a systematic analysis for the global burden of Disease Study 2015. Lancet (London England).

[17] Collaborators GO (2017). Health effects of overweight and obesity in 195 countries over 25 years. N Engl J Med.

[18] Xu P, Huang Y, Hou Q, Cheng J, Ren Z, Ye R (2022). Relationship between physical activity and mental health in a national representative cross-section study: its variations according to obesity and comorbidity. J Affect Disord.

[19] Okati-Aliabad H, Ansari-Moghaddam A, Kargar S, Jabbari N. Prevalence of obesity and overweight among adults in the middle east countries from 2000 to 2020: a systematic review and meta-analysis. Journal of Obesity. 2022;2022. 10.1155/2022/8074837 PMC8831052 35154826

[20] Liu Q, He H, Yang J, Feng X, Zhao F, Lyu J (2020). Changes in the global burden of depression from 1990 to 2017: findings from the Global Burden of Disease study. J Psychiatr Res.

[21] Ng M, Fleming T, Robinson M, Thomson B, Graetz N, Margono C (2014). Global, regional, and national prevalence of overweight and obesity in children and adults during 1980–2013: a systematic analysis for the global burden of Disease Study 2013. Lancet.

[22] Prentice AM, Jebb SAJO (2001). Beyond body mass Index.

[23] Kahn HS, Bullard KMJTA. Beyond body mass index: advantages of abdominal measurements for recognizing cardiometabolic disorders. 2016;129(1):74–81. e2. 10.1016/j.amjmed.2015.08.010 PMC5292922 26302146

[24] Krakauer NY, Krakauer, JCJPo. A new body shape index predicts mortality hazard independently of body mass index. 2012;7(7):e39504. 10.1371/journal.pone.0039504 PMC3399847 22815707

[25] Maessen MF, Eijsvogels TM, Verheggen RJ, Hopman MT, Verbeek AL, de Vegt, FJPo. Entering a new era of body indices: the feasibility of a body shape index and body roundness index to identify cardiovascular health status. 2014;9(9):e107212. 10.1371/journal.pone.0107212 PMC4167703 25229394

[26] He S, Chen XJP (2013). Could the new body shape index predict the new onset of diabetes mellitus in the. Chin Population?.

[27] Cheung YBJPO. A body shape index in middle-age and older Indonesian population: scaling exponents and association with incident hypertension. 2014;9(1):e85421. 10.1371/journal.pone.0085421 PMC3893209 24454862

[28] Bergman RN, Stefanovski D, Buchanan TA, Sumner AE, Reynolds JC, Sebring NG (2011). Better Index body Adiposity.

[29] Ehrampoush E, Arasteh P, Homayounfar R, Cheraghpour M, Alipour M, Naghizadeh MM et al. New anthropometric indices or old ones: which is the better predictor of body fat? 2017;11(4):257–63. 10.1016/j.dsx.2016.08.027 27578617

[30] Zhao D, Li Y, Zheng L, Yu KJA. Brief communication: body mass index, body adiposity index, and percent body fat in asians. 2013;152(2):294–9. 10.1002/ajpa.22341 23996556

[31] Sung Y-A, Oh J-Y, Lee HJY. Comparison of the body adiposity index to body mass index in Korean women. 2014;55(4):1028. 10.3349/ymj.2014.55.4.1028 PMC4075363 24954333

[32] Appelhans BM, Kazlauskaite R, Karavolos K, Janssen I, Kravitz HM, Dugan S et al. How well does the body adiposity index capture adiposity change in midlife women? The SWAN fat patterning study. 2012;24(6):866–9. 10.1002/ajhb.22330 PMC3640421 23015468

[33] Johnson W, Chumlea WC, Czerwinski SA, Demerath EWJO. Concordance of the recently published body adiposity index with measured body fat percent in european-american adults. 2012;20(4):900–3. 10.1038/oby.2011.346 PMC3988697 22095112

[34] Sun G, Cahill F, Gulliver W, Yi Y, Xie Y, Bridger T et al. Concordance of BAI and BMI with DXA in the Newfoundland population. 2013;21(3):499–503. 10.1002/oby.20009 23404962

[35] Schulze M, Thorand B, Fritsche A, Häring H, Schick F, Zierer A et al. Body adiposity index, body fat content and incidence of type 2 diabetes. 2012;55(6):1660–7. 10.1007/s00125-012-2499-z 22349074

[36] Lichtash CT, Cui J, Guo X, Chen Y-DI, Hsueh WA, Rotter JI et al. Body adiposity index versus body mass index and other anthropometric traits as correlates of cardiometabolic risk factors. 2013;8(6):e65954. 10.1371/journal.pone.0065954 PMC3679008 23776578

[37] Geliebter A, Atalayer D, Flancbaum L, Gibson CDJO. Comparison of body adiposity index (BAI) and BMI with estimations of% body fat in clinically severe obese women. 2013;21(3):493–8. 10.1038/oby.2012.187 PMC3470730 23592658

[38] Freedman DS, Thornton JC, Pi-Sunyer FX, Heymsfield SB, Wang J, Pierson RN Jr et al. The body adiposity index (hip circumference ÷ height1. 5) is not a more accurate measure of adiposity than is BMI, waist circumference, or hip circumference. 2012;20(12):2438–44. 10.1038/oby.2012.81 PMC3477292 22484365

[39] Gibson C, Atalayer D, Flancbaum L, Geliebter AJI. Body adiposity index (BAI) correlates with BMI and body fat pre-and post-bariatric surgery but is not an adequate substitute for BMI in severely obese women. 2012;10(1):9. PMC3520094 23243391

[40] Bagger YZ, Tankó LB, Alexandersen P, Qin G, Christiansen C (2004). The implications of body fat mass and fat distribution for cognitive function in elderly women. Obes Res.

[41] Kerwin DR, Zhang Y, Kotchen JM, Espeland MA, Van Horn L, McTigue KM (2010). The cross-sectional relationship between body Mass Index, Waist–Hip ratio, and cognitive performance in Postmenopausal Women enrolled in the women’s Health Initiative. J Am Geriatr Soc.

[42] Hughes T, Borenstein A, Schofield E, Wu Y, Larson E (2009). Association between late-life body mass index and dementia: the Kame Project. Neurology.

[43] Kuo HK, Jones RN, Milberg WP, Tennstedt S, Talbot L, Morris JN (2006). Cognitive function in normal-weight, overweight, and obese older adults: an analysis of the advanced cognitive training for independent and vital elderly cohort. J Am Geriatr Soc.

[44] Lotfi K, Hassanzadeh Keshteli A, Saneei P, Afshar H, Esmaillzadeh A, Adibi P (2022). A body shape index and body roundness index in relation to anxiety, depression, and psychological distress in adults. Front Nutr.

[45] Hadi S, Momenan M, Cheraghpour K, Hafizi N, Pourjavidi N, Malekahmadi M (2020). Abdominal volume index: a predictive measure in relationship between depression/anxiety and obesity. Afr Health Sci.

[46] Sajjadi H, Harouni GG, Rafiey H, Vaez-Mahdavi M, Vameghi M, Kamal SHMJJPM (2020). Contextual and Individual Determinants of Mental Health: a cross-sectional Multilevel Study in Tehran. Iran.

[47] Asghari A, Saed F, Dibajnia PJIJ (2008). Psychometric properties of the Depression anxiety stress Scales-21 (DASS-21) in a non-clinical. Iran Sample.

[48] SAMANI S, JOUKAR B. A study on the reliability and validity of the short form of the depression anxiety stress scale (DASS-21). 2007.

[49] Kulkarni P, Nagendra N, Ashok N, Sunil KD, Siddalingappa H, Madhu B. World Health Organization-Body Mass Index for Age Criteria as a Tool for Prediction of Childhood and adolescent morbidity: a Novel Approach in Southern Karnataka, India. Int J Prev Med. 2014;5(6). PMC4085921 25013688

[50] Choi HS, Cho YH, Lee SY, Park EJ, Kim YJ, Lee JG et al. Association between new anthropometric parameters and arterial stiffness based on brachial-ankle pulse wave velocity. 2019;12:1727. 10.2147/DMSO.S211542 PMC6731955 31564940

[51] Moghaddam MB, Aghdam FB, Jafarabadi MA, Allahverdipour H, Nikookheslat SD, Safarpour S (2012). The Iranian version of International Physical Activity Questionnaire (IPAQ) in Iran: content and construct validity, factor structure, internal consistency and stability. World Appl Sci J.

[52] Benjamini Y, Hochberg Y (1995). Controlling the false discovery rate: a practical and powerful approach to multiple testing. J Roy Stat Soc: Ser B (Methodol).

[53] de Wit L, Have Mt, Cuijpers P, de Graaf R (2022). Body Mass Index and risk for onset of mood and anxiety disorders in the general population: results from the Netherlands Mental Health Survey and Incidence Study-2 (NEMESIS-2). BMC Psychiatry.

[54] Brumpton B, Langhammer A, Romundstad P, Chen Y, Mai X-M (2013). The associations of anxiety and depression symptoms with weight change and incident obesity: the HUNT study. Int J Obes.

[55] Eik-Nes TT, Tokatlian A, Raman J, Spirou D, Kvaløy K. Depression, anxiety, and psychosocial stressors across BMI classes: a Norwegian population study-the HUNT study. Front Endocrinol. 2022:1848. 10.3389/fendo.2022.886148 PMC9399822 36034441

[56] Zedler B, von Lengerke T, Emeny R, Heier M, Lacruz ME, Ladwig K-H (2013). Obesity and symptoms of depression and anxiety in pre-and postmenopausal women: a comparison of different obesity indicators. Psychother Psychosom Med Psychol.

[57] Arroyo KJ, Ramos-Torres G, Mezones-Holguin E, Blümel JE, Barón G, Bencosme A (2018). Association between waist-to-height ratio and anxiety in middle-aged women: a secondary analysis of a cross-sectional multicenter latin American study. Menopause.

[58] Bruffaerts R, Demyttenaere K, Vilagut G, Martinez M, Bonnewyn A, De Graaf R (2008). The relation between body mass index, mental health, and functional disability: a European population perspective. Can J Psychiatry.

[59] Grundy A, Cotterchio M, Kirsh VA, Kreiger N (2014). Associations between anxiety, depression, antidepressant medication, obesity and weight gain among Canadian women. PLoS ONE.

[60] Ma W, Yan Z, Wu W, Li D, Zheng S, Lyu J. Dose-Response Association of Waist-to-Height ratio plus BMI and risk of Depression: evidence from the NHANES 05–16. Int J Gen Med. 2021:1283–91. 10.2147/IJGM.S304706 PMC8055360 33883926

[61] Luppino FS, de Wit LM, Bouvy PF, Stijnen T, Cuijpers P, Penninx BW (2010). Overweight, obesity, and depression: a systematic review and meta-analysis of longitudinal studies. Arch Gen Psychiatry.

[62] Gariepy G, Nitka D, Schmitz N (2010). The association between obesity and anxiety disorders in the population: a systematic review and meta-analysis. Int J Obes.

[63] Capuron L, Su S, Miller AH, Bremner JD, Goldberg J, Vogt GJ (2008). Depressive symptoms and metabolic syndrome: is inflammation the underlying link?. Biol Psychiatry.

[64] Eckel RH, Grundy SM, Zimmet PZ (2005). The metabolic syndrome. Lancet.

[65] Whitmer R, Gustafson D, Barrett-Connor E, Haan M, Gunderson E, Yaffe K (2008). Central obesity and increased risk of dementia more than three decades later. Neurology.

[66] Avelar TM, Storch AS, Castro LA, Azevedo GV, Ferraz L, Lopes PF (2015). Oxidative stress in the pathophysiology of metabolic syndrome: which mechanisms are involved?. Jornal Brasileiro De Patologia E Med Laboratorial.

[67] Milaneschi Y, Simmons WK, van Rossum EF, Penninx BW (2019). Depression and obesity: evidence of shared biological mechanisms. Mol Psychiatry.

[68] Penninx BW, Milaneschi Y, Lamers F, Vogelzangs N (2013). Understanding the somatic consequences of depression: biological mechanisms and the role of depression symptom profile. BMC Med.

[69] Vreeburg SA, Zitman FG, van Pelt J, DeRijk RH, Verhagen JC, van Dyck R (2010). Salivary cortisol levels in persons with and without different anxiety disorders. Psychosom Med.

[70] Prunty A, Clark MK, Hahn A, Edmonds S, O’Shea A (2020). Enacted weight stigma and weight self stigma prevalence among 3821 adults. Obes Res Clin Pract.

[71] Sutin AR, Stephan Y, Luchetti M, Aschwanden D, Strickhouser JE, Lee JH (2021). BMI, weight discrimination, and the trajectory of distress and well-being across the Coronavirus Pandemic. Obesity.

[72] Spoorthy MS, Pratapa SK, Mahant S (2020). Mental health problems faced by healthcare workers due to the COVID-19 pandemic–A review. Asian J Psychiatry.

