# Supplementary material for: Dose-response association of obesity and risk of mental health among tehranian residents: result of a cross-sectional study
Source: BMC Public Health. 2024 May 29;24:1444. doi: 10.1186/s12889-024-18670-z (PMC11138087; doi:10.1186/s12889-024-18670-z)
Supplement: Supplementary file 1 — Supplementary Material 1 [file 12889_2024_18670_MOESM1_ESM.docx]

**Supplementary Table 1**: Anthropometric measure ranges classified by tertiles

| **BMI** (kg/m²) | | | **WHR** | | | **WHtR** | | | **ABSI** (m^11/6^kg^-2/3^) | | | **BAI** (%) | | |
| --- | --- | --- | --- | --- | --- | --- | --- | --- | --- | --- | --- | --- | --- | --- |
| **Lowest** | **Middle** | **Highest** | **Lowest** | **Middle** | **Highest** | **Lowest** | **Middle** | **Highest** | **Lowest** | **Middle** | **Highest** | **Lowest** | **Middle** | **Highest** |
| ≤ 25.28 | 25.29 – 28.62 | ≥ 28.63 | ≤ 0.84 | 0.85 – 0.92 | ≥ o.93 | ≤ 0.52 | 0.53 – 0.58 | ≥ 0.59 | ≤ 0.077 | 0.078 – 0.081 | ≥ 0.082 | ≤ 27.75 | 27.76 – 32.86 | ≥ 32.87 |

**Commands for the dose-response analyses**

* Continuous outcomes

label var dose "Dose (grams/d)"

label var n "Participants"

sort id category

gen max=.

bysort id: replace max=_N

qui summarize id

global maxn=r(max)

display $maxn

gen md=.

gen semd=.

bysort id: replace md=meanbmd-meanbmd[1]

bysort id: replace semd=sqrt(((sdbmd[1]^2)/n[1])+((sdbmd^2)/n))

replace semd=0 if category==1

bysort id (category) : replace md=0.0001 if md[_n] == 0 & semd[_n]!=0

gen type=4

gen lci=md - invnorm(0.975) * semd

gen uci=md + invnorm(0.975) * semd

* Non-linear

capture drop doses1

capture drop doses2

sum dose, d

mkspline doses = dose, nk(3) cubic displayknots

mat knots = r(knots)

drmeta md doses1 doses2, se(semd) data(n sdbmd) id(id) type(type)

testparm doses1 doses2

return list

global overp=r(p)

display $overp

if $overp<0.001 {

global overp="< 0.001"

}

else {

global overp: display %5.3f $overp

global overp="= $overp"

}

display "$overp"

testparm doses2

return list

global pdep=r(p)

display $pdep

if $pdep<0.001 {

global pdep="< 0.001"

}

else {

global pdep: display %5.3f $pdep

global pdep="= $pdep"

}

display "$pdep"

drmeta md doses1 doses2, se(semd) data(n sdbmd) id(id) type(type)

capture drop fity

capture drop efity

predict fity, xb

gen efity = exp(fity)

twoway (line efity dose, sort lc(red))

global xtitle="xtitle(BMI (kg/m2))"

global doserange= "dose(15(1)40)"

global ytitle="ytitle(Mean)"

global yscale="yscale(range($ymin $ymax)) ylabel(-8 -6 -4 -2 0 2 4 6 , format(%5.3g))"

global yline="yline(0, lcol(red) lw(thick) lp(.))"

global xscale="xscale(range(15 40))"

global xlabel="xlabel(15 20 25 30 35 40)"

global scheme="scheme(s1color)"

global t1title="title(Anxiety)"

drmeta md doses1 doses2, se(semd) data(n sdbmd) id(id) type(type)

drmeta_graph, matk(knots) ref(19.18) $xtitle $doserange $ytitle $yscale $yline $xscale $xlabel $scheme $t1title

graph addplot scatter md dose if category==1, mfcolor(gs13) mlcolor(gs10) ms(|) below jitter(0.1) $yscale $xscale $xlabel

graph addplot scatter md dose[w=1/semd^2] if category!=1, mcolor(gs13) ms(oh) below $yscale $xscale $xlabel note(" " "P{sub:dose-response} $overp; P{sub:non-linearity} $pdep", size(small))

graph export hipbmd-nonlinear1.pdf, replace
